# A Sequential Framework for Improving Identifiability of FE Model Updating Using Static and Dynamic Data

**DOI:** 10.3390/s19235099

**Published:** 2019-11-21

**Authors:** Sehoon Kim, Namgyu Kim, Young-Soo Park, Seung-Seop Jin

**Affiliations:** 1 Research Institute for Infrastructure Performance, Korea Infrastructure Safety & Technology Corporation, Jinju 52856, Korea; sehoon.kim@kistec.or.kr; 2 Department of Civil and Environmental Engineering, Sejong University, Seoul 05006, Korea; namgyu.kim@sejong.ac.kr; 3 Department of Infrastructure and Safety Research, Korea Institute of Civil Engineering and Building Technology, Goyang 10223, Korea; youngsoopark@kict.re.kr; 4 Sustainable Infrastructure Research Center, Korea Institute of Civil Engineering and Building Technology, Goyang 10223, Korea

**Keywords:** finite element model updating, heterogeneous data, parameter compensation, parameter identifiability, sequential framework

## Abstract

By virtue of the advances in sensing techniques, finite element (FE) model updating (FEMU) using static and dynamic data has been recently employed to improve identification on updating parameters. Using heterogeneous data can provide useful information to improve parameter identifiability in FEMU. It is worth noting that the useful information from the heterogeneous data may be diluted in the conventional FEM framework. The conventional FEMU framework in previous studies have used heterogeneous data at once to compute residuals in the objective function, and they are condensed to be a scalar. In this implementation, it should be careful to formulate the objective function with proper weighting factors to consider the scale of measurement and relative significances. Otherwise, the information from heterogeneous data cannot be efficiently utilized. For FEMU of the bridge, parameter compensation may exist due to mutual dependence among updating parameters. This aggravates the parameter identifiability to make the results of the FEMU worse. To address the limitation of the conventional FEMU method, this study proposes a sequential framework for the FEMU of existing bridges. The proposed FEMU method uses two steps to utilize static and dynamic data in a sequential manner. By using them separately, the influence of the parameter compensation can be suppressed. The proposed FEMU method is verified through numerical and experimental study. Through these verifications, the limitation of the conventional FEMU method is investigated in terms of parameter identifiability and predictive performance. The proposed FEMU method shows much smaller variabilities in the updating parameters than the conventional one by providing the better predictions than those of the conventional one in calibration and validation data. Based on numerical and experimental study, the proposed FEMU method can improve the parameter identifiability using the heterogeneous data and it seems to be promising and efficient framework for FEMU of the existing bridge.

## 1. Introduction

Structural deterioration under external conditions (e.g., traffic loading) degrades the performance of the bridge along with time. It is important to evaluate the conditions of the existing bridges and assess their performances under potential scenarios (e.g., earthquake). In this context, finite element (FE) model has been widely used for the condition assessment. Typically, an initial FE model lacks in representing the real behaviors of the existing bridges due to the deterioration and imprecise model parameters (stiffness and boundary conditions) in FE models. As a result, it is crucial for successful prognostic analysis to represent the current condition of the bridge by improving the accuracy of the FE model. FE model updating (FEMU) is such a process to associate FE models with corresponding existing bridges based on experimental data. This process is an inverse problem of identifying updating parameters (i.e., model parameters) by refining an initial FE model using experimental data [1,2].

FEMU employs an iterative optimization scheme of adjusting model parameters by minimizing residuals between measured and computed reference properties. The reference properties can be categorized into modal properties from dynamic data (e.g., natural frequency and mode-shape) and static data (e.g., displacement and strain). Conventionally, the modal properties have been widely used for FEMU, since modal properties can be readily extracted from ambient vibration tests [3,4]. The FEMU using natural frequencies and mode-shapes have been applied to suspension bridges [5,6], cable-stayed bridges [3,7], pre-stressed concrete girder bridges [8,9,10] and steel-box girder bridge [11]. To improve the results of FEMU, model flexibility [12] has been also investigated. As sensing techniques for static data become more advanced [13,14], static data such as displacement and strain can be measured more reliably. As a result, the static data has been investigated to the FEMU [15,16,17]. Hereafter, the dataset with a single type of experimental data is referred to as homogeneous data.

It is worth noting that FEMU using homogeneous data (only modal or static data) has some limitations as follows: (1) homogeneous data cannot provide sufficient information in the case that model parameters are inter-dependent to the experimental data (i.e., parameter compensation), so that it may pose FEMU problems to an ill-posed problem; and (2) the accuracy of the response in extrapolation cannot be ensured. For example, FEMU based on modal data cannot ensure the accuracy of the static responses. As a result, a compulsory FEMU results in a wrong FE model based on residual minimization (i.e., minimization of objective function using optimization). Therefore, the updated FE model should be validated using independent data to evaluate the validity of the FEMU result.

In FEMU of bridges, it is very important to identify the stiffness of the superstructures (e.g., girder and cross beam) and support conditions for representing the dynamic and static behavior of the bridge. Since both the stiffness of the superstructures and support conditions are mutually dependent (i.e., parameter compensation), it is not easy to guarantee the identifiability using homogeneous data. This can result in similar responses from a different set of the updating parameters. Such a problem regarding to identifiability is referred to as equifinality [18]. The way of alleviating equifinality is to utilize additional and useful information by exploring different types of the experimental data. In this context, there have been several works to use both modal and static data for the FEMU [19,20,21]. Hereafter, the dataset with different types of the experimental data is referred to as heterogeneous data.

When both modal and static data are simultaneously employed into FEMU, they are combined into a scalar by a residual sum to quantify the goodness-of-fit of model parameters for heterogeneous data. Using heterogeneous data at once can dilute the useful information from each type of the data. For example, a certain type of heterogeneous data (having large scale) can dominate the residual sum in the objective function. Therefore, it is important to balance their influences by assigning proper weighting factors and need special cares for formulating the objective function. Unfortunately, the optimal weighting factors and the formulation of the objective function are case-dependent and they may be achieved by the trial-and-error method. Despite its importance, the previous studies have employed the heterogeneous data at once [19,20,21] without careful formulation of the objective function.

To deal with the abovementioned issues in using heterogeneous data, this study proposes a simple and efficient framework for FEMU of the bridge. The main contributions of this study are summarized as follows: (1) this study shows the limitation of using all heterogeneous data at once under parameter compensation in FEMU of the bridge and (2) a sequential FEMU method using two-step is proposed to overcome the limitation and improve parameter identifiability. The rationale behind the proposed FEMU method is to elicit useful information from the heterogeneous data separately in a sequential framework. In the first step, static data is used to identify the boundary condition using neural network [18]. Then, identified values of boundary conditions are fixed and stiffness of the superstructure is updated through dynamic FEMU. By doing so, the parameter compensation in FEMU can be addressed and the useful information from the heterogeneous data can be utilized. Consequently, the results of the FEMU are significantly improved with consistent identifiability of the model parameters.

The rest of this paper is organized as follows. Section 2 presents the existing FEMU and the proposed FEMU. In Section 3, the proposed FEMU method is evaluated using a numerical example of the plate girder bridge. Section 4 shows the field experiment of the real bridge and evaluates the proposed FEMU method. In the numerical study and experimental study using field data, the proposed FEMU method was evaluated with the existing FEMU for heterogeneous data (modal and static data). Lastly, the conclusion and further studies are presented in Section 5.

## 2. Research Backgrounds

This section provides overviews of the conventional and proposed FEMU methods for bridges. For simplicity, this study employed a deterministic FEMU based on residual minimization using global optimization. However, these FEMU could be applied to other non-deterministic FEMU such as Bayesian inference. We first presented sensing techniques to measure the heterogeneous data such as the modal property, deflection and rotational angle at supports. Then, the conventional FEMU was presented with its limitations for using the heterogeneous data. Lastly, the sequential framework for FEMU was proposed to improve the parameter identifiability.

### 2.1. Heterogeneous Sensor Systems for Modal and Static Data

Heterogeneous data in previous studies for FEMU includes modal properties [2,3,9,12], deflection including strain [13,15,16,21]. The modal properties, deflection and strain with a load test have been widely used, while the rotational angle at supports has been recently investigated [14,22,23]. The rotational angle at supports can provide the information on the support conditions and this has the significant influence on both static and dynamic behaviors of the bridge. The measurement system for heterogeneous data is illustrated in Table 1.

Gyro sensors are one of the representative sensors to measure the angular speed at the supports—the velocity of the rotational angle [22]. However, using the angular speed is limited to FEMU of the bridge, since the precisions of the gyro sensors are not sufficiently accurate to measure the small response of the rotational angle at the support. Most bridges have a very small response at the support except for the long-span bridges such as suspension and cable-stayed bridges. On the other hand, inclinometers can measure the rotational degrees at supports directly. However, it is not suitable to measure the rotational angle at the support of the bridge, since it has a poor resolution and accuracy to measure the rotational angles less than 3 × 10^−3^ degree.

In this context, vision-based systems have received much attention, since dynamic and static displacements can be measured using the camera and target with reasonable accuracy and sampling rate [13,14,23]. In addition, displacements at targets can be converted into rotational angle at support with the known distance from the support to target panel [14,23]. In a real application of the vision-systems for the bridge, the vision-based systems are easily affected by external conditions such as weather and illumination. These systems work properly only when installed in the correct line of camera sight. In addition, the distance from the camera to the target panel affects the measurement accuracy. In this study, the vision-based system was adopted to measure the rotational angle of bridges [14]. The vision-based system for rotational angle is composed of a laser source, a frame grabber and a camcorder. In order to produce the point in a target panel, the green laser module was used with the wavelength of 532 nm, divergence of 0.5 m·rad and operating distance of 50 m. The frame grabber has a resolution of 640 pixels × 480 pixels with the capture speed of 30 frames per second. For the camera, a commercially available home video camcorder (Sony pj340 (https://www.sony.com/electronics/support/memory-camcorders-hdr-pj-series/hdr-pj340)) was used.

**Figure 1:**
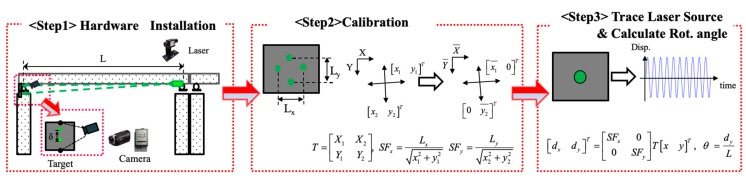
Measurement procedures for a dynamic rotational angle.

The rotational angle is calculated from the displacement obtained by image processing techniques [13]. The laser is firmly attached at the bottom of the bridge girder or a bridge deck at support. The target panel is installed at a bridge pier or an abutment on the other side, which is regarded as a fixed location. The target should be installed perpendicular to the laser beam. The distance between the laser and target can be pre-determined. The target size with four white spots can be determined by considering the actual size of target area, which can be traced by a camera with a telescopic lens. To convert the pixel information to actual displacement, a target image with four spots is captured. The horizontal and vertical lengths (*L_x_*, *L_y_*) can be determined by considering the maximum displacement of the laser source. A transformation matrix and scaling factors can be calculated using direction vectors and the actual size of the target. When a moving vehicle runs over the bridge, the support will rotate together with the attached laser pointer. The projected laser on the target can be traced by a camcorder, while the displacement can be calculated by the computed scaling factors and a transformation matrix. Finally, the rotational angle at the support can be calculated using the measured displacement divided by the distance from the laser source to target. The accuracy of the system was verified from laboratory tests [14] and field test [23]. In this system, the noise level for the distance up to 30 m is found to be around $1.8\;\times \;10^{-4}$ degree. The vision-based rotational angle measurement system and its application procedure are illustrated in Figure 1.

**Figure 2:**
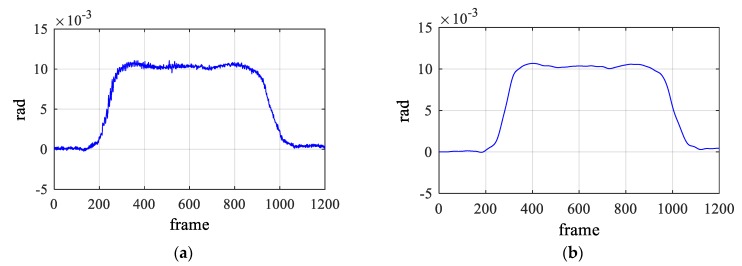
Preprocessing of rotational angle using a low-pass filter. (**a**) Raw data and (**b**) filtered data.

The measured static data contains high frequency components due to measurement noise, so that a third-order Butterworth filter was applied as a low-pass filter with the cut-off frequency of 1 Hz to remove the measurement noise. It is clearly seen in Figure 2 that the noise components was removed successfully using the filter and the only static component remains.

In order to reliably construct the FE model of the bridges, more advanced sensing techniques are required for measuring various dynamic and static data. In the case of measuring dynamic data, sufficient excitation is the most important to extract more modes with reliable and accurate mode-shapes. In this context, it is necessary to develop more efficient ways of exciting the bridges without blocking the traffics [24]. For the computer-vision system, the reliability of the static data is reduced due to the illumination of the target panel and data drift caused by the fixation of the camera. To address the illumination, recent advance in the image processing techniques is introduced to reduce the effect of light [25]. One way of addressing these issues is to indirectly estimate displacement by fusing acceleration data and strain data [26]. In this context, advances in the sensing technique for FEMU should be required to improve the accuracy and reliability of the heterogeneous data.

### 2.2. Conventional FE Model Updating Using One-Stage Framework

In FEMU for bridges, model parameters are typically parameterized as updating parameters to identify the stiffness of superstructures and boundary condition (e.g., support and abutment). These model parameters have the influence on the dynamic and static behavior of the bridges. When these model parameters are selected as updating parameters for FEMU, the mutual dependence between them may exist. The mutual dependence can result in the similar objective function values with different combination of the updating parameters. For example, the support condition is modeled by the spring element to represent the support condition ranging from movable to fixed condition [22,27,28]. When updating FE models with stiffness of the superstructure and spring elements at the support, “increasing the stiffness of the superstructures with decrease of the spring constant” can give similar effects in the objective function to “decreasing the stiffness of the superstructures with increase of the spring values”. This mutual dependence is termed as parameter compensation, and it aggravates the parameter identifiability in FEMU. One remedy for the parameter compensation is to provide additional and useful information on updating parameters.

Conventional FEMU using heterogeneous data (modal and static data) has been investigated by previous studies for FEMU of the bridges [19,20,21]. In these studies, modal and static data were used for the objective function. The residual in the objective function can be defined as a squared sum of the difference between the heterogeneous data and corresponding predictions. In the FEMU, updating parameters are identified by minimizing the residual sum (i.e., objective function) and it can be interpreted as the minimization problem in optimization. General formulation for the objective function is defined as:

(1) $$J=\alpha \sum_{i=1}^{N}w_{i}^{f}\sqrt{{\left(\frac{f_{i}^{EXP}-f_{i}^{FEM}}{f_{i}^{EXP}}\right)}^{2}}+\beta \sum_{i=1}^{N}w_{i}^{\Phi }\left(1-MAC\left(\Phi_{i}^{EXP},\Phi_{i}^{FEM}\right)\right)+\Upsilon \sum_{j=1}^{M}\omega_{j}^{\gamma }\sqrt{{\left(\frac{y_{j}^{EXP}-y_{j}^{FEM}}{\;y_{j}^{EXP}}\right)}^{2}}\;,$$

where N is the number of the identified modal properties; $f_{i}^{FEM}$ and $f_{i}^{EXP}$ denote the *i*-th natural frequencies from the FE model and experiment, respectively; $MAC\left(\Phi_{i}^{EXP},\Phi_{i}^{FEM}\right)$ is the modal assurance criterion (MAC) value between the *i*th mode-shapes from the FE model ($\Phi_{i}^{FEM}$) and experiment ($\Phi_{i}^{EXP}$); $\omega_{i}^{f}$ and $\omega_{i}^{\Phi }$ are the weighting factors for the residuals of the natural frequencies and mode-shapes to impose the relative significance of each quantity, where ($\sum \omega_{i}^{f}=\sum \omega_{i}^{\emptyset }=1$); *M* is the number of the measured static responses $y_{i}^{FEM}$ and $y_{i}^{EXP}$ denote the *i*th static responses from the FE model and experiment, respectively and $\omega_{j}^{\gamma }$ is the weighting factors for *i*th static responses ($\sum \omega_{i}^{\gamma }=1$). α, β and γ are weighting factors for each responses, where 0≤α,β,γ≤1 and α+β+γ=1.

**Figure 3:**
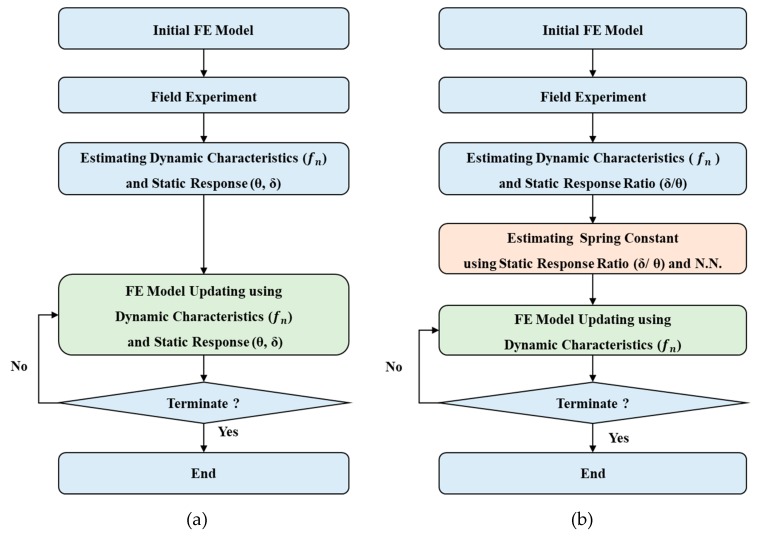
Flowchart for the FEMU methods. (**a**) The conventional FEMU method using modal and static data simultaneously and (**b**) the proposed FEMU method.

When heterogeneous data is simultaneously used for the FEMU, the objective function should be carefully formulated with well-balanced weighting factors. However, the optimal weighting factor is not known in advance and they can be achieved by trial-and-error method. A certain type of the residuals can be dominated in the objective function with improper weighting factors, so that the objective function cannot evaluate the goodness-of-fit of each residual properly to identify the correct values of the updating parameters. The flowchart for the conventional FEMU method is illustrated in Figure 3a.

### 2.3. Proposed FE Model Updating Using Sequential Framework

To address the abovementioned limitations, a sequential framework for FEMU is proposed in this study. Boundary conditions can significantly affect the responses of the bridge, so that the updating parameter for the boundary conditions is typically considered in FEMU. Considering that the existing bridges generally exhibit the deterioration of the support conditions, the identification of the support conditions is necessary. There are several studies that consider the effect of the boundary conditions in FEMU. In these studies, the boundary condition was considered as the important updating parameter in FEMU [1,7,23]. The underlying reasons for using the sequential framework are as follows: (1) parameter compensation can be alleviated by separating updating parameters in a sequential manner; and (2) a set of well-balanced weighting factors is not required in objective function, so that the issues regarding weighting factors can be avoided. By doing that, the sequential framework for FEMU of the bridge can significantly improve the parameter identification. The sequential framework proposed in this study was performed as the following two steps for FEMU:

1. Boundary condition identification using a neural network (N.N.) with static data (1st step): static data (i.e., displacement (δ) and rotational angle of the support (θ)) is used to identify the boundary condition. The boundary condition is a model by rotational spring constant at support, and the rotational spring constant is set to represent the support condition from the movable support to fixed support. The rotational spring constant is identified using our previously proposed FEMU method [23]. To learn the relationship between the static data and support condition, the N.N. is trained with the ratio of the static data from the spring constant calculated from the initial model. Once the N.N. is trained and validated, the rotational spring constant is identified from the ratio of the measured static data the N.N. technique.
2. FEMU using modal data (2nd step): Once the rotational spring constants at support are identified, FEMU using modal data is performed to identify the stiffness of the superstructure. In the second step, the identified values of the rotational spring constant are fixed during this step. The objective function in this step is shown in Table 2.
3. Validity evaluation of the updated FE model using validation data: To diagnose the predictive performance of the updated FE model, it is evaluated using the validation data in the extrapolation (i.e., not used in the objective function).

The flowchart for the proposed FEMU method is illustrated in Figure 3b.

## 3. Numerical Verification

This section evaluated the proposed FEMU method using the numerical study with the FE model of the plate girder bridge. The conventional FEMU method was also performed to compare its performance with that of the proposed FEMU method. There are two reasons for using this simple FE model: (1) the limitation of the conventional FEMU method can be investigated by using the updating parameters having the mutual dependence (two spring constants at supports and Young’s modulus of the bridge members such as girder, slab, and cross beam); and (2) the performance of two methods can be easily evaluated using identification results of the true values of the updating parameters.

### 3.1. Preliminary Work for FE Model Updating

**Figure 4:**
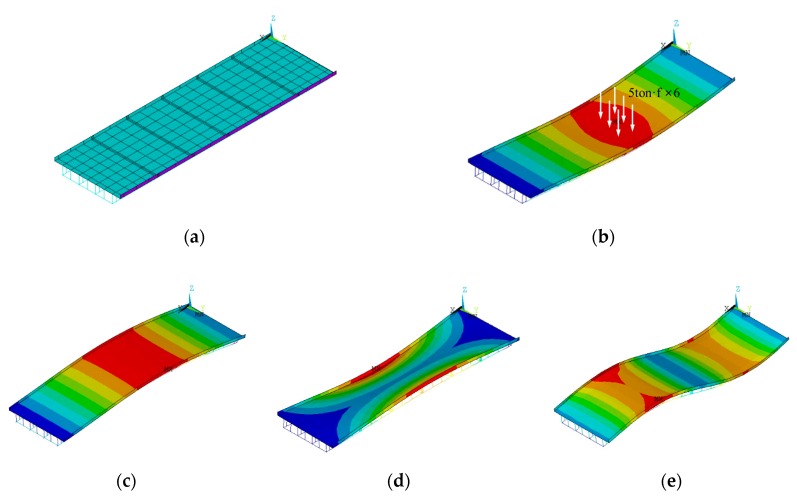
Numerical simulation using the FE model of the plate-girder bridge. (**a**) FE model of the test bridge; (**b**) static load analysis; (**c**) 1st bending mode; (**d**) 1st torsion mode and (**e**) 2nd bending mode.

The FE model consists of 162 beam elements and 318 shell elements with 535 nodes. The FE model was constructed in the commercial FE software (i.e., ANSYS Mechanical APDL). Dynamic analysis was performed by modal analysis to compute the modal properties, while static analysis with 6 points load of 5 ton·f was simulated to compute static data (e.g., displacement at center and rotational angles at support). The geometry of the FE model and the FE analysis results are described in Figure 4.

For updating parameters in FEMU, four model parameters were chosen as: (1) rotational spring constant at the left support (*K_A_*), (2) rotational spring constant at the left support (*K_B_*), (3) Young’s modulus of the main girder (*K_G_*) and (4) Young’s modulus of the slab and cross beam (*K_G_*, *K_S_* and *K_C_*). Since the slab and the cross beam are related to the lateral load distribution performance of bridges, they were grouped as one updating parameter. To generate synthetic data, the true values of these updating parameters were used as tabulated in Table 3. The modal properties and static data are also shown in Figure 4b–e. To emphasize the limitation of the conventional FEMU method (i.e., parameter compensation and dilution of the information), the target data in Table 4 were injected by additive Gaussian noise to account for the presence of the measurement noise. Since the noise levels of the modal properties are relatively smaller than the static data (deflection and rotational angle) in reality, the modal properties were perturbed by Gaussian noise to have 80 dB of signal-to-noise ratio (SNR). On the other hand, the Gaussian noises for the static data were generated with the high-noise level (SNR = 60 dB). With different random seeds, five synthetic data was generated.

Based on perturbation of the nominal values in Table 3, the upper and lower bounds were determined as: (1) the range of the rotational spring constants ($K_{A}$ and $K_{B}$) was set between 1 × 10^7^ N·m/rad and 1 × 10^13^ N·m/rad based on the change of the natural frequencies, since the outside of this range does not change the natural frequencies; and (2) to actuate the parameter compensation, the range between upper and lower bound for *K_G_*, *K_S_* and *K_C_* were determined from 0.3 to 1.5 (i.e., −70%–50%).

### 3.2. FE Model Updating for Numerical Verification

The proposed FEMU using sequential framework was performed. In the first step, the rotational spring constants at the supports were identified using the N.N. with the ratio of the static data. In order to construct the N.N., 200 training samples were randomly generated for each repetition. It is worth noting that space-filling design such as optimized Latin-hypercube sample or quasi-random sequence can suppress the variability in the N.N. construction. However, this study intentionally generated the training samples using random sampling to evaluate the robustness to the variability in the construction of the N.N. The N.N. was constructed using an input layer with two nodes ($\delta /\theta_{A}$ and $\delta /\theta_{B}$), two hidden layers (five and three nodes for each layer, respectively) and an output layer with two nodes (*K_A_* and *K_B_*). The training samples were split into 70% for training data sets, 15% for validation data sets. The remaining 15% of the training samples was used for test data sets to evaluate the generalized predictions of the N.N. As a global optimizer, the genetic algorithm (G.A.) [29] was used to identify the updating parameters for the conventional FEMU method and the second step in the proposed FEMU method. G.A. was employed by MATLAB with 100 generations. The other hyper-parameters for G.A. were used by the default setting. The conventional and proposed FEMU were performed for the five synthetic data.

Based on the objective function in Table 2, the conventional FEMU was performed to identify the true values of the updating parameters. The results of the updating parameter were tabulated in Table 3 with their summary statistics (e.g., mean and coefficient of variation (C.V.)). The updating result shows that (1) the relative error of the rotational spring constant was up to 26% from the true value on average (i.e., mean) and (2) the relative error of the stiffness of members were up to 3% from the true value.

In the proposed method, the rotational spring constants were firstly estimated using the ratio of the static data. Based on the results from N.N. (Table 3), the relative errors of both best and mean value were less than 2% with the C.V. of 0.02. This implies that the values of the rotational spring constant were well identified to the true values. Once the rotational spring constants were identified, the FEMU was performed using modal data (natural frequency and MAC value) to identify the stiffness of the bridge members (*K_G_*, *K_S_* and *K_C_*). In this step, the rotational spring constants were fixed to the identified value from each estimation in the first step. The relative errors of all updating parameters were less than 1% from the true value, and the C.V. was computed less than 0.02. Table 4 shows the updating results of the modal and static data. The relative errors of these data were significantly improved than those of the conventional FEMU method.

The proposed FEMU method provides more accurate values of the updating parameters than those of the conventional FEMU method. In addition, the conventional FEMU method generally provides the larger values of the C.V. than those of the proposed FEMU method. These results reveal that the parameter identifiability can be enhanced using the heterogeneous data in the sequential framework rather than using them at once (i.e., one-stage).

## 4. Experimental Verification through a Field Test

### 4.1. Target Bridge and Field Experiment

**Figure 5:**
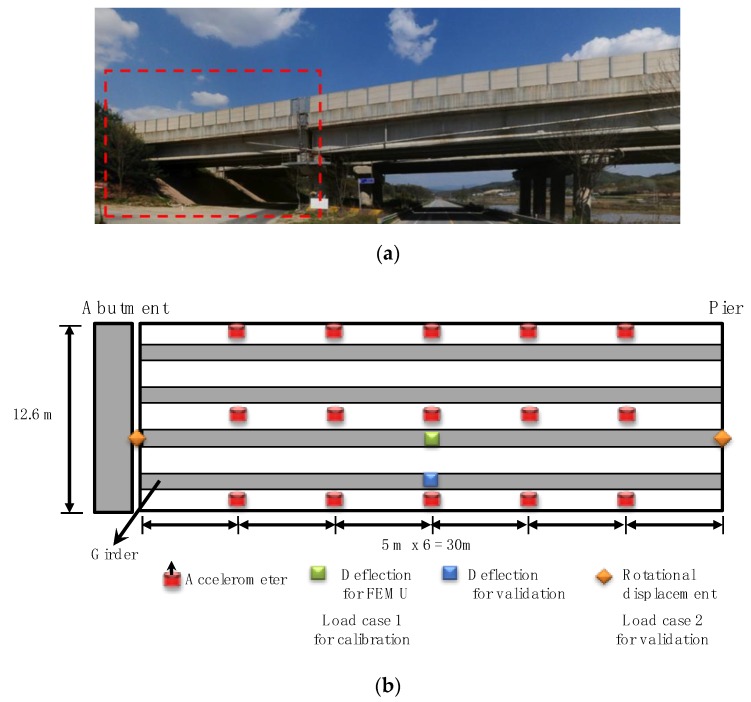
Test bridge and sensor configuration. (**a**) target span; (**b**) measurement system configuration and loading positions of load cases.

The field test of the existing bridge was used for verification of the proposed FEMU method. The test bridge was a pre-stressed concrete bridge with the length of 30 m and width of 12.6 m. The bridge consisted of a concrete slab and four main girders with three cross-beams and two diaphragms, as shown in Figure 5. Fifteen accelerometers were installed to measure the vibration for modal identification, while the static data was measured by the four cameras using two lasers and four targets as presented in Section 2.1 and Figure 1. The sensor configuration is shown in Figure 5.

**Figure 6:**
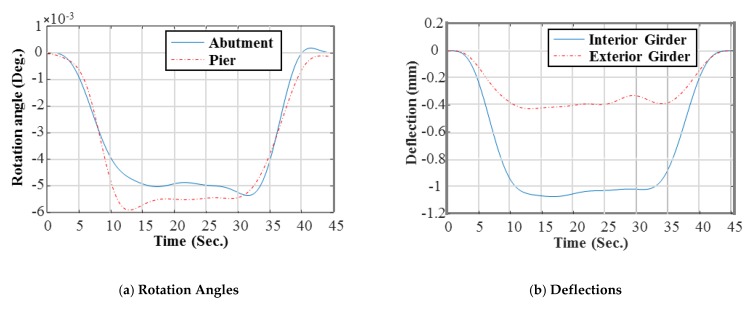
Rotation angles and deflections measurements of L.C. 1: calibration data. (**a**) Rotation angles: avg. value at the abutment bound = 4.40 × 10^−3^ deg. Avg. value at the pier bound = 5.38 × 10^−3^ deg. and (**b**) deflections: avg. value at the interior girder = 0.99 mm. Avg. value at the exterior girder = 0.43 mm.

**Figure 7:**
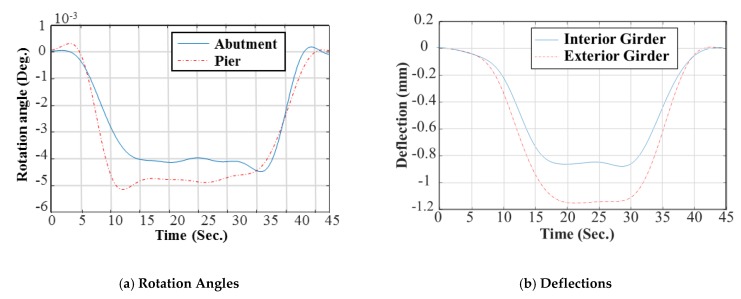
Rotation angles and deflections measurements of L.C. 2: validation data. (**a**) Rotation angles avg. value at the abutment bound = 3.92 × 10^−3^ deg. Avg. value at the pier bound = 4.66 × 10^−3^ deg. and (**b**) deflections: avg. value at the interior girder = 0.82 mm. Avg. value at the exterior girder = 1.03 mm.

To induce the static deflection and rotational angle at supports, loading tests were conducted using the truck with the weight of 25.78 ton·f. The rotational angles at supports were measured with a laser using the cameras and targets installed on each support [14], while another camera and target were installed at the abutment and the center of the main girder to measure the deflection of the bridge [13]. To obtain the static data (rotational angle and deflection), two load cases were performed as: (1) the first load case is to obtain the static data for calibration data (Load Case 1 in Figure 5b; and (2) the validation data was measured using Load Case 2 in Figure 9e to evaluate validity of the updated FE model (i.e., extrapolation in the prediction). These load cases were repeated by six times for averaging. A set of the filtered static data was averaged and the results are plotted in Figure 6 and Figure 7.

**Figure 8:**
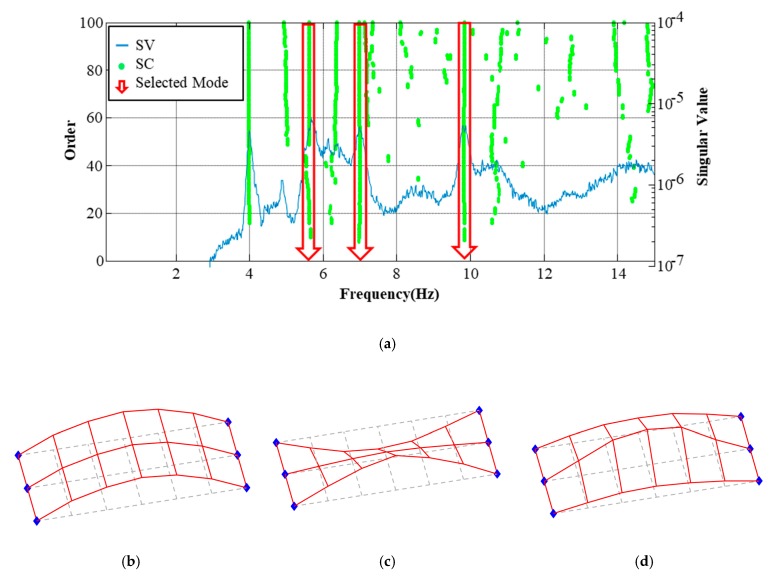
Extracted modal properties. (**a**) Selects modes from the stabilization chart (SC) and SVD result; (**b**) bending mode: 5.63 HZ; (**c**) torsional mode: 7.00 Hz and (**d**) transverse bending mode: 9.84 Hz.

Fifteen accelerometers with a 5-by-3 array were installed on the bridge deck to extract the modal properties including vertical bending, torsional and lateral bending mode. The ambient vibration test was conducted for 3 hours with the sampling frequency of 100 Hz. The measured accelerations were analyzed using both stochastic subspace identification (SSI) [30] and frequency domain decomposition (FDD) [31]. Stabilization chart (SC) of the SSI was plotted with singular values (SV) of FDD in Figure 8a. Three modes were identified as shown in Figure 8b–d.

### 4.2. Preliminary Work for Experimental Verification 

**Figure 9:**
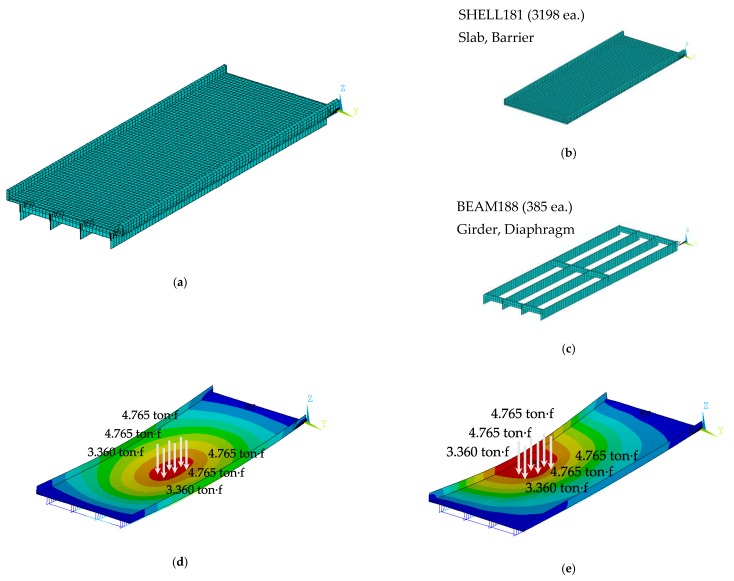
FE model of a pre-stressed bridge. (**a**) FE model of the test bridge; (**b**) shell element of the FE model; (**c**) beam element of the FE model; (**d**) Load Case 1 for calibration and (**e**) Load Case 2 for validation.

ANSYS Mechanical APDL was used to construct an initial FE model based on construction drawings. Girder and cross beams were modeled using beam elements, while concrete slabs and barrier were modeled using shell elements. To represent the deterioration of the supports, a 1-D rotational spring element was used for constraint the rotation of the boundary condition and it was applied at the nodes of both ends. Figure 9 shows an initial FE model with two load cases for calibration and validation data.

Since the identified modes include the vertical bending, torsional and lateral bending mode, the stiffness of the superstructures were chosen to improve the accuracy of these modes. On the other hand, the rotational spring constant were considered to represent the support conditions. In this context, five updating parameters were selected as: (1) two rotational spring constants at supports (2EA), (2) the relative stiffness ratio of the girder (1EA), (3) the relative stiffness ratio of the main slab (1EA) and (4) the relative stiffness ratio of the cross beam (1EA). The details of the updating parameters are shown in Table 5.

The change of the natural frequencies according to the change of the rotational spring constant was linear. The support condition acts as a movable end (i.e., the roller) below 1 × 10^7^ times of the nominal value (1 N·m/rad) with constant natural frequencies, and the natural frequencies linearly increase between 1 × 10^7^ and 1 × 10^14^ times of the nominal value. The natural frequencies converged to the specific values over 1 × 10^14^ times of the nominal values by changing support condition into the fixed end. Based on these observations, the ranges of the rotational spring constant were set between 1 × 10^7^ and 1 × 10^14^ N·m/rad. To consider the contribution of the tendons in the girders on the stiffness, the upper bounds for relative stiffness ratio were set to 2.0 times to the nominal value (34.13 GPa). The lower bounds of the updating parameters were set to 0.3 times of the nominal value based on the following reasons: (1) there may be a reduction in the stiffness of the cross-beam due to the imperfect connectivity between the girder and cross beam; and (2) this aggravates the parameter compensation among the updating parameters to investigate the limitation of the conventional method. In this context, the range of the relative stiffness ratio was between 0.3 and 2.0.

To quantify the relative contributions of the updating parameters for the target responses, Fourier amplitude sensitivity testing (FAST) was used [32] as a global sensitivity analysis. The relative stiffness ratio of the girder mainly contributed to the output variance in the vertical bending and torsional modes (*f*1 and *f*2) and deflection of internal girder (*δ_Int_*). The relative stiffness ratios of both slab and cross-beam generally had an influence on the modal data. Especially in the lateral bending mode (*f3*), the relative stiffness ratio of the slab was the largest influential one among the updating parameters. For deflection of the internal girder, the only influential parameter was the relative stiffness of the girder. The rotational spring constants were the most influential parameters for the rotational angle at each support, and other parameters seems to be non-influential. Based on these observations, five model parameters were sufficiently influential to update the initial FE model using the measured modal and static data.

### 4.3. FE Model Updating for Field Experimental Verification

The FEMU was performed using both the conventional and proposed method. The G.A. was employed to update the FE model using 100 generations with default hyper-parameters. The FEMU was performed by 10 repetitions. In this experimental study, the mode-shape was not considered from the objective function in Table 2. In ambient vibration tests, mode-shapes are easily contaminated by the low signal-to-noise ratio, insufficient excitation and so on. In addition, the mode-shapes were not intentionally used to aggravate the identifiability of the updating parameters. As a result, the mode-shape was used only for mode pairing.

**Figure 10:**
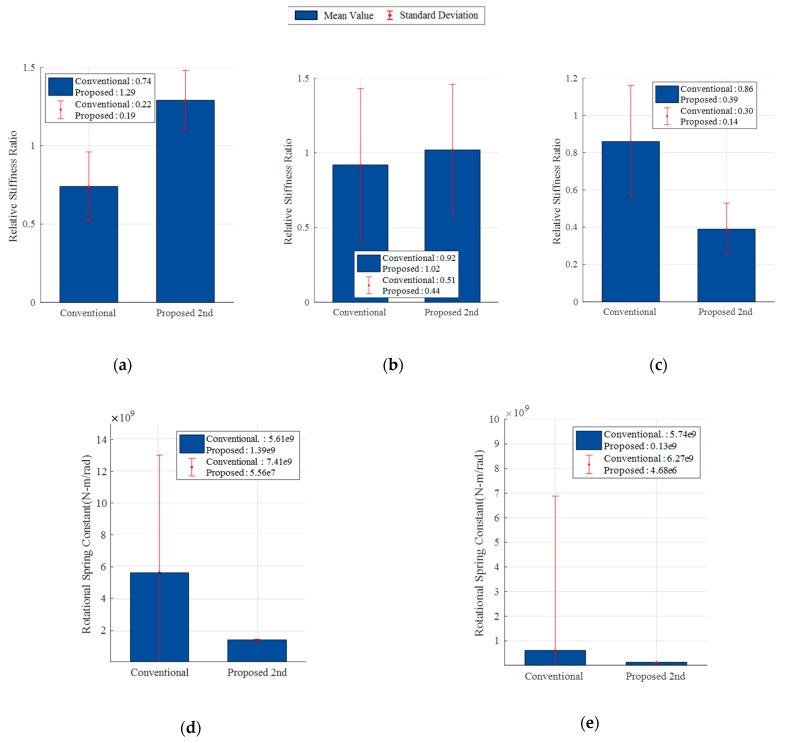
Model updating result of model parameters. (**a**) *K_G_*; (**b**) *K_S_*; (**c**) *K_C_*; (**d**) *K_A_* and (**e**) *K_P_*.

In the conventional FEMU of the one-stage framework, the heterogeneous data was used simultaneously as: (1) three static responses of load case 1 (*δ_Int_*, *θ_Abutment_* and *θ_Pier_*) and (2) first three natural frequencies (*f*1, *f*2 and *f*3). Figure 10 shows the updated results and their summary statistics through 10 repetitions. The results show that (1) the relative error of the deflection and natural frequencies are less than 7% from those of the experimental data; and (2) the relative error of the both rotation angles are up to 17%. Compared to the initial FE model, the conventional FEMU method improves the static and dynamic behaviors of the FE model. However, the coefficient of variation (C.V.) of the relative stiffness ratio of the girder and rotational spring constant were estimated to be 0.61–1.32. This implies that there is the parameter compensation between these two updating parameters. Stated differently, the large values of the C.V. indicate a lack of the parameter identifiability under the identical experimental data. Since the additional stiffness of the tendon in the girders was not considered in the FE modeling, it was expected that the updated value of the girder increased from the nominal value. However, the relative stiffness ratio of the girder was identified near 74% of the nominal value and this updating result seemed to be unrealistic.

**Figure 11:**
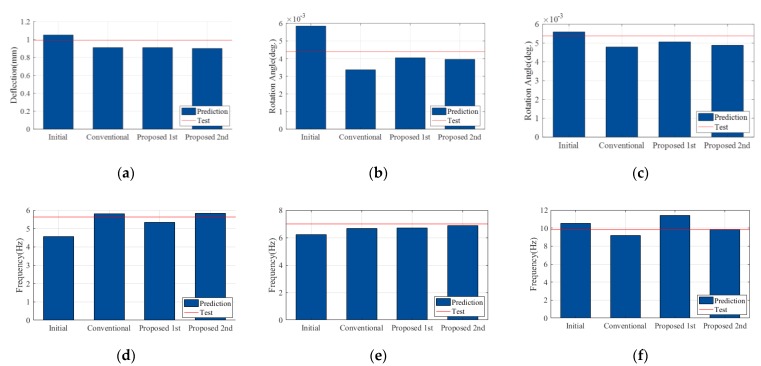
Comparison of the updated result for the calibration data. (**a**) *δ_Int_* of *LC*1; (**b**) *θ_Abutment_* of *LC*1; (**c**) *θ_pier_* of *LC*1; (**d**) *f*_1_; (**e**) *f*_2_ and (**f**) *f*_3_.

**Figure 12:**
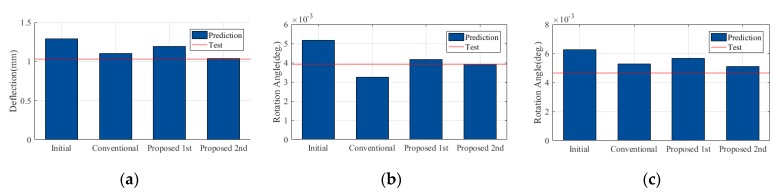
Comparison of the updated result for the validation data. (**a**) *δ_Ext_* of *LC*2; (**b**) *θ_Abutment_* of *LC*2 and (**c**) *θ_pier_* of *LC*2.

The proposed FEMU using sequential framework consists of two steps. In the first step, the rotational spring constants were estimated using the N.N. and measured ratio of the static data from the field experiment. The architecture of N.N. and splitting data are identical to those in the numerical verification. Based on the results from 10 repetitions, the best identified value and their summary statics were computed. The values of the rotational springs were identified as shown in Figure 10d–e with the C.V. less than 0.04. As shown in Figure 11, the updated FE model from the first step was not sufficient to represent the target static and dynamic behaviors yet. To improve the accuracy of the updated FE model from the first step, FEMU was performed using modal data to identify the updating parameters of the superstructures. In this step, the rotational spring constants were fixed to the identified value from each repetition. As shown in Figure 10, the C.V. of the relative stiffness ratios were computed between 0.14 and 0.44, which were less than those of the conventional FEMU method. This indicates that the proposed FEMU method improves the parameter identifiability the smaller C.V. than those of the conventional method. As expected, the updated value of the girder increases around 130% of the nominal value due to effects of the tendons. As shown in Figure 11, the relative errors of all target data are less than 10%. This indicates that the updated FE model from the proposed FEM method is better calibrated than that of the conventional FEMU method. Figure 12 compares the accuracy for the validation data (i.e., static responses of load case (2) from each method. The proposed FEMU method only provides less than 10% of the relative errors for the validation data. This indicates that the proposed FEMU method is much more reliable to improve the extrapolation predictions (validation data) than the conventional FEMU method.

Through the experimental verification, it could be concluded that the proposed FEMU method could improve the parameter identifiability using the sequential framework. As a result, the updated responses for both calibration and validation data were significantly improved with much lower parameter variability (i.e., smaller C.V.). Based on these results, it could be concluded that the proposed FEMU method could provide better results of the FEMU against the conventional one.

## 5. Conclusions

As sensing techniques become more advanced, various types of the experimental data can be measured. As a result, various responses from the infra-structures are available using the accelerometer, optical fiber sensing and vision-based systems. The heterogeneous data including static and modal data can provide informative information for structural behaviors and using the heterogeneous data for FEMU has been investigated by the previous study to improve the updating results. In this context, using the heterogeneous data can alleviate the parameter compensation among the updating parameter by providing additional information on updating parameters.

Conventionally, FEMU employs both static and modal data simultaneously to formulate the objective function (i.e., one-stage approach). When the static and modal data is simultaneously used to formulate the objective function, FEMU should be carefully performed to maximize the utilization of information in the heterogeneous data and avoid the certain type of the responses dominates the residual sum in the objective function. In this context, there are two goals of this study as follows: (1) this study shows the limitation of the conventional FEMU method through the numerical and experimental study; and (2) The FEMU method using a new sequential framework is proposed to address the abovementioned limitations and improve the parameter identifiability. The proposed FEMU method consists of two steps; firstly, the proposed FEMU method identifies the rotational spring constants at the support condition using static data and N.N. Once the rotational spring constants are identified, they are fixed to the identified values and FEMU using modal properties is performed to identify the remaining updating parameters (e.g., stiffness of the superstructures). By doing so, parameter compensation can be minimized by identifying the updating parameters separately; (2) the information from static and modal data can be independent utilized and (3) the optimal weighting factor does not needed to balance relative significances between modal and static data. The proposed FEMU method was evaluated by numerical and experimental study with the conventional FEMU method. Based on the results, the following summaries could be drawn as:

- In the numerical study, the proposed FEMU method identified more accurate values of the updating parameters under the presence of the noise. As a result, the proposed method provided the better predictive performances over calibration and validation data than those of the conventional FEMU method.
- In the experimental verification, the conventional FEMU method generally provided larger values of the C.V. than those of the proposed FEMU methods. This large C.V. indicates a lack of the parameter identifiability due to parameter compensation. In addition, the identified values from the proposed method were more realistic than those of the conventional method (i.e., additional stiffness by the tendon in the girders).
- For calibration data in experimental verification, the proposed FEMU method provided more accurate predictions than those of the conventional FEMU method. In addition, more accurate predictions in extrapolation were also observed. This implies that the proposed FEMU method identified the accurate updating parameters and improved the static and dynamic behaviors of the existing structure.

The parameter identifiability could be enhanced using the heterogeneous data sequentially rather than using them at once. The proposed FEMU method could update the initial FE model accurately, so that the updated FE model from the proposed FEMU method could improve the reliability for predictive performances for further applications (e.g., condition and risk assessments using the updated FE model).

## Figures and Tables

**Table 1:** Measurement systems for heterogeneous data for finite element model updating (FEMU).

Sensor	Specification	Performance
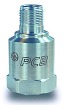	Accelerometer (PCB 393B12, PCB Piezotronics, NY, USA) (https://www.pcb.com/products?model=393B12)	
	Sensitivity	10,000 mV/g
	Measurement range	0.5 g·pk
	Frequency Range	0.15–1000 Hz
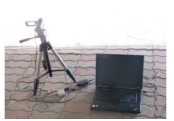	Vision-based displacement measurement sensor [13]	
	Measuring range	40 m
	Resolution	0.0042 mm/pixel (at 30 m)
	Sampling rate	30 Hz, 60 Hz
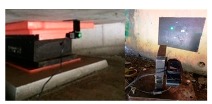	Vision-based rotational angle measurement sensor [14]	
	Measuring range	40 m
	Resolution	1.8 × 10^−4^ degree (at 30 m)
	Sampling rate	30 Hz, 60 Hz

**Table 2:** Comparison of FEMU methods.

Method		Date for FEMU		Objective Function
		Static	Modal	
Conventional method		O	O	$\alpha \sum_{i=1}^{N}w_{i}^{f}{\left(\frac{f_{i}^{EXP}-f_{i}^{FEM}}{f_{i}^{EXP}}\right)}^{2}+\beta \sum_{j=1}^{M}\omega_{j}^{MAC_{j}}\frac{1-\sqrt{MAC_{j}^{2}}}{\;MAC_{j}}+\Upsilon \sum_{k=1}^{M}\omega_{k}^{y}{\left(\frac{y_{k}^{EXP}-y_{k}^{FEM}}{\;y_{k}^{EXP}}\right)}^{2}$
Proposed method	1st step	O	-	N.N.
	2nd step	-	O	$\sum_{i=1}^{N}w_{i}^{f}{\left(\frac{f_{i}^{EXP}-f_{i}^{FEM}}{f_{i}^{EXP}}\right)}^{2}+\sum_{j=1}^{M}\omega_{j}^{MAC_{j}}\frac{1-\sqrt{MAC_{j}^{2}}}{\;MAC_{j}}$

**Table 3:** Relative error of updating parameters.

Model	$K_{A}\;(N\text{·}m/\mathrm{rad})$			$K_{B}\;(N\text{·}m/\mathrm{rad})$			*K*_*G*_ (Ratio ^(^^1^^)^)			*K*_*S*_ and *K*_*C*_ (Ratio)		
	Best	Mean	C.V.	Best	Mean	C.V.	Best	Mean	C.V.	Best	Mean	C.V.
Target	3.00 × 10^8^	-	-	8.00 × 10^9^	-	-	0.90	-	-	0.80	-	-
Conventional method (relative error) ^(2)^	3.02 × 10^8^ (0.50%)	2.22 × 10^8^ (−25.86%)	0.49	8.03 × 10^9^ (0.39%)	8.14 × 10^9^ (1.75%)	0.02	0.94 (4.74%)	0.93 (2.91%)	0.03	0.73 (−8.70%)	0.80 (−0.05%)	0.09
Proposed Method (relative error)	3.09 × 10^8^ (1.17%)	3.09 × 10^8^ (1.17%)	0.01	7.94 × 10^9^ (−0.71%)	7.94 × 10^9^ (−0.71%)	0.02	0.90 (−0.21%)	0.90 (0.35%)	0.01	0.80 (0.25%)	0.80 (−0.42%)	0.02

^(1)^ Ratio: $\frac{\mathrm{Target}\;\mathrm{Stiffness}}{\mathrm{Nominal}\;\mathrm{Stiffness}}$, ^(2)^ relative error: $\frac{\mathrm{Updated}-\mathrm{Target}}{\mathrm{Target}}\times 100$ (%).

**Table 4:** Comparison of predictive performance.

Model	1st Bending (Hz)	1st Torsion (Hz)	2nd Bending (Hz)	δ_C_/θ_A_ (mm/rad)	δ_C_/θ_B_ (mm/rad)
Target (before perturbed) *	5.13 (5.14)	5.54 (5.55)	15.60 (15.61)	9.80 (9.82)	33.10 (33.11)
Conv. (relative error)	5.16 (0.58%)	5.54 (0.06%)	15.42 (−1.15%)	9.95 (1.53%)	33.11 (0.03%)
Prop. (relative error)	5.13 (−0.04%)	5.54 (−0.03%)	15.60 (−0.06%)	9.84 (0.39%)	33.04 (−0.18)

* Before perturbed: response of FE model before noise injected.

**Table 5:** Updating parameters.

Parameter (Notation)	Change in Variable	Initial Value (Nominal Value)	Range
Rotational Spring Constant at Abutment (*K_A_*)	Linear	1 (1 N·m/rad)	1 × 10^7^–1 × 10^14^ (Movable–Fixed)
Rotational Spring Constant at Pier (*K_P_*)		1 (1 N·m/rad)	
Relative Stiffness Ratio of Girder (*K_G_*)	Linear	1 (34.13 GPa)	0.3–2.0
Relative Stiffness Ratio of Slab (*K_S_*)	Linear	1 (34.13 GPa)	0.3–2.0
Relative Stiffness Ratio of Cross beam (*K_C_*)	Linear	1 (34.13 GPa)	0.3–2.0
